# Phosphorylation of H3-Thr3 by Haspin Is Required for Primary Cilia Regulation

**DOI:** 10.3390/ijms22147753

**Published:** 2021-07-20

**Authors:** Roberto Quadri, Sarah Sertic, Anna Ghilardi, Diego Rondelli, Guido Roberto Gallo, Luca Del Giacco, Marco Muzi-Falconi

**Affiliations:** 1 Istituto Nazionale di Genetica Molecolare, Via Sforza 35, 20122 Milano, Italy; 2 Dipartimento di Bioscienze, Università degli Studi di Milano, Via Celoria 26, 20133 Milano, Italy; sarah.sertic@unimi.it (S.S.); anna.ghilardi@unimi.it (A.G.); diego.rondelli@unimi.it (D.R.); guido.gallo@unimi.it (G.R.G.); luca.delgiacco@unimi.it (L.D.G.)

**Keywords:** Haspin, primary cilia, Dido3, HDAC6, ciliopathy, H3T3, zebrafish

## Abstract

Primary cilia are commonly found on most quiescent, terminally differentiated cells and play a major role in the regulation of the cell cycle, cell motility, sensing, and cell–cell communication. Alterations in ciliogenesis and cilia maintenance are causative of several human diseases, collectively known as ciliopathies. A key determinant of primary cilia is the histone deacetylase HDAC6, which regulates their length and resorption and whose distribution is regulated by the death inducer-obliterator 3 (Dido3). Here, we report that the atypical protein kinase Haspin is a key regulator of cilia dynamics. Cells defective in Haspin activity exhibit longer primary cilia and a strong delay in cilia resorption upon cell cycle reentry. We show that Haspin is active in quiescent cells, where it phosphorylates threonine 3 of histone H3, a known mitotic Haspin substrate. Forcing Dido3 detachment from the chromatin prevents Haspin inhibition from impacting cilia dynamics, suggesting that Haspin activity is required for the relocalization of Dido3–HDAC6 to the basal body. Exploiting the zebrafish model, we confirmed the physiological relevance of this mechanism. Our observations shed light on a novel player, Haspin, in the mechanisms that govern the determination of cilia length and the homeostasis of mature cilia.

## 1. Introduction

Mitosis, when the genetic material is precisely segregated between daughter cells, is an extremely delicate phase of the cell cycle. Failures in this process may generate aneuploidies leading to cell death and diseases (e.g., cancer). Thus, an intricate network of specialized proteins exists to ensure that all M-phase events occur in a precise and ordered way.

Haspin is an atypical protein kinase whose most characterized role is the mitotic phosphorylation of histone H3 on Thr3 (H3-Thr3) [1] which is reversed in a PP1/Repo-Man dependent manner at the end of mitosis [2]. Phosphorylated H3-Thr3 is bound by Survivin [3], a component of the chromosomal passenger complex (CPC), which is required to ensure proper alignment of the chromatids on the metaphase plate [1,4]. Accordingly, loss of H3-Thr3 phosphorylation results in chromosomal segregation defects [1,5]. Other roles for Haspin have been reported in the coupling of cell-cycle progression to polarization dynamics [6,7,8,9], chromatid cohesion [10,11], organization of microtubule organizing centers (MTOCs) during murine meiosis [12], and asymmetrical histone inheritance [13] and migration [14], and Haspin inhibitors have a well-established anticancer activity [14,15].

The trimethylation of lysine 4 of histone H3 (H3-K4me_3_ hereafter) is a docking site for proteins bearing a plant homeodomain (PHD) finger [16]. PHD proteins are dispersed from the chromatin upon phosphorylation of H3-Thr3 and H3-Thr6 (by Haspin and protein kinase Cβ_I_, respectively) [17]. Among the proteins that bind H3-K4me_3_ is the death inducer-obliterator (Dido), a protein whose three identified isoforms have a role in stemness regulation [18,19], mitotic spindle organization, cell differentiation [19,20], alternative splicing [21], and cilia dynamics [22]. Consistently with the observed inhibitory effect of H3-Thr3 phosphorylation on PHD domain binding to H3-K4me_3_, phosphorylation of H3-Thr3 in mitosis abolishes the binding of the isoform 3 of Dido (Dido3 hereby) to H3-K4me_3_ in vitro, promoting its relocalization to the mitotic spindle [20].

Primary cilia are microtubular structures that protrude from the surface of most cells of the human body, where they receive a variety of signals and act as key determinants in tissue development and homeostasis [23]. From a structural point of view, primary cilia are composed of nine radial microtubular doublets and differ from motile cilia for the absence of the two central doublets that allow motility. Ciliary microtubules are subjected to a plethora of specialized modifications, among which acetylation is the most common, and deacetylation of ciliary tubulin by HDAC6 results in ciliary shortening and resorption [24]. The axoneme, the framework of the cilium, originates at the end of mitosis from a modified centriole differentiated to a basal body. Once matured, the basal body is not capable to sustain a mitotic apparatus so that most cells that exhibit primary cilia in the human body are quiescent, G_0_ cells [25], and an intricate network exists to prevent detrimental ciliogenesis in mitotic cells [26,27,28]. Indeed, in vertebrates, ciliogenesis is mutually exclusive with cell division. Before cells undergo the G1/S transition, the first wave of cilia resorption is observed, and the complete disassembly occurs before mitotic entry. Thus, mitotic cells are devoid of primary cilia. Commitment to a new cell cycle promotes ciliary resorption through binding of HEF1 to Aurora A [29], which in turn triggers the activity of HDAC6 at the basal body [30]. There, HDAC6 targets acetylated tubulin and, by doing so, restricts ciliary length and facilitates their resorption [29,30].

Thus far, a role for primary cilia has been described in processes ranging from cell-cycle regulation (by sequestering the MTOC of the cell) [28], sensing [31], DNA damage response [32], embryological development [33], and autophagy [34]. As it acts against cell-cycle commitment by sequestering the MTOC as a basal body, loss of cilia is commonly observed in different (though not all) tumors [35,36]. Moreover, defects in primary cilia are related to several pathological conditions encompassing mental retardation, impaired brain plasticity, cystic kidney disease, obesity, and other developmental malformations, collectively known as ciliopathies [37].

In this context, Dido3 acts to mediate the recruitment of the histone deacetylase HDAC6 to the basal bodies of primary cilia [22]. Underlying the major contribution of Dido3 in HDAC6 distribution and primary cilia regulation, a Dido3∆NT that lacks the DNA-binding domain shows a predominant basal body accumulation, along with HDAC6, leading to a reduction in ciliary length [22].

The possibility that H3-Thr3 is phosphorylated in quiescent cells and on primary cilia regulation have not been elucidated.

As previously mentioned, Haspin activity has been observed and predominantly studied during mitosis or meiosis, although being present during the whole cell cycle. Its activity is regulated through an autoinhibitory domain that folds on the catalytic domain, inhibiting the enzyme. Haspin activity is promoted by a series of specific mitotic phosphorylation by CDK and PLK1 [38,39] that reposition the autoinhibitory domain.

Here, we show that Haspin is active in G_0_ and demonstrate an unexpected role of Haspin in this cell cycle stage. The activity of the kinase is required to modulate primary cilia dynamics by regulating the correct distribution of Dido3 and HDAC6. We show that H3-Thr3 is phosphorylated in quiescent cells and that loss of this modification causes longer and more persistent cilia in a Dido3-dependent manner. Finally, we extend these results to zebrafish embryos, providing functional insights of loss of Haspin-mediated cilia regulation in a whole living organism.

## 2. Results

### 2.1. Haspin Phosphorylates H3-Thr3 during Quiescence

The atypical protein kinase Haspin has been described in several eukaryotic organisms and has been shown to be expressed in proliferating cells. During mitosis, Haspin phosphorylates histone H3-Thr3 to build a scaffold for CPC recruitment, ensuring proper alignment of chromosomes on the metaphase plate [1,5]. Haspin is predicted to be locked in an inactive conformation during interphase (G_1_, S, and G_2_ phases); PLK1-dependent phosphorylation relieves Haspin auto-inhibition [38]. To investigate possible functions of Haspin outside of mitosis, we analyzed Haspin in quiescent cells (G_0_).

**Figure 1:**
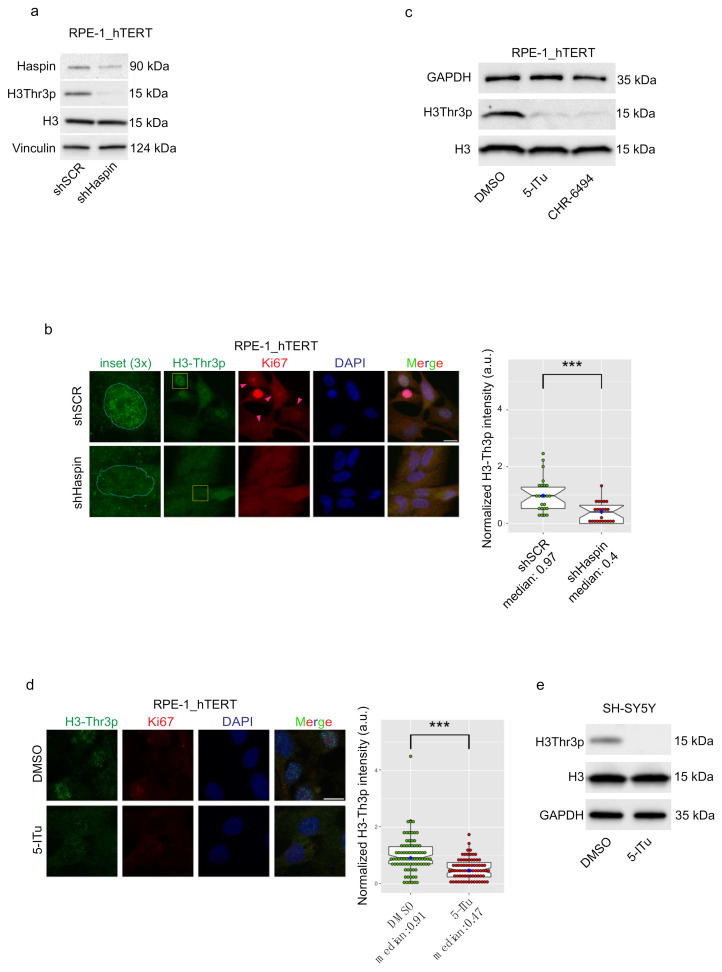
Haspin is active in G_0_ phase. (**a**) RPE-1_hTERT cells stably silenced with shSCR (control) or shHaspin were seeded and harvested after 48 h serum starvation to induce entry in G_0_. Silencing efficiency and H3-T3 phosphorylation were monitored by Western blotting; (**b**) immunofluorescence against Ki67 (proliferation marker) and H3-T3 in shSCR and shHaspin cells serum-starved for 48 h. Histone phosphorylation was quantified and is reported to the right. Silencing control is shown in Supplementary Figure S1e; (**c**) RPE-1_hTERT cells were seeded and serum-starved for 48 h, then treated for a further 24 h with DMSO or Haspin inhibitor 5-ITu (10 nM) and CHR-6494 (50 nM); H3T3p was monitored by Western blotting; (**d**) cells treated with 5-ITu were processed for immunofluorescence against Ki67 and H3-T3. Histone phosphorylation was quantified and is reported to the right; (**e**) Western blot analysis monitoring the levels of H3-T3p in SH-SY5Y that were differentiated into neuron-like cells, as described in Material and Methods, and then incubated for 24 h with 10 nM 5-ITu. Graphs in b, d show the median abundance of phosphorylated H3-Thr3; boxes include 50% of the data points, notch represent confidence interval (median ±1.58 IQR/sqrt(n)). *t*-test was applied as a statistical measurement, n.s.; not significant, *** *p* < 0.005. Scale bars in b, d: 20 µm.

Control (shCTR) or Haspin-silenced (shHaspin) RPE-1_hTERT cells were harvested after 48 h of serum starvation to stabilize G_0_ permanency (Supplementary Information, Figure S1a for FACS profile), and we analyzed phosphorylated H3-Thr3. We found that H3-Thr3 was phosphorylated in quiescent cells, and this modification was strongly reduced upon silencing of Haspin (Figure 1a, silencing efficiency measured by Western blotting: 62%; the control in exponentially growing cells is shown in Figure S1b,c, silencing efficiency measured by a Western blotting: 73%). It is noteworthy that in agreement with previous reports showing a peak of Haspin activity in mitosis, H3-Thr3p levels are much higher in exponentially growing cells than in noncycling cultures (Supplementary Information, Figure S1d). These data clearly indicate that at least a fraction of Haspin is active in noncycling cells.

To exclude a possible contribution of residual cycling cells within the quiescent population, we analyzed H3-Thr3 phosphorylation in single cells by immunofluorescence, using Ki67 as a marker of cycling cells. Intriguingly, as shown in Figure 1b, most of the G_0_ control cells (red arrows) were positive for H3-Thr3 phosphorylation. Haspin silencing (Supplementary Information, Figure S1e, silencing efficiency measured by a Western blot: 92%) confirmed that in G_0_, phosphorylation of H3-Thr3 relies on Haspin (Figure 1b).

The specific Haspin inhibitors 5-iodotubercidine (5-ITu) [40], and CHR-6494 have been previously employed to characterize Haspin functions. We thus exploited such drugs to dissect Haspin’s impact on G_0_. To this end, we first established a working concentration sufficient to inhibit the kinase without impacting the survival of exponentially growing cells. Treatment with 10nM 5-ITu or 50 nM CHR-6494 blocks most Haspin kinase activity on H3-Thr3 with minimal effects on cell viability (Figure 1c and Supplementary Information, Figure S1f). The drugs were added for 24 h to cells (pre-starved for 48 h). In agreement with the results obtained by downregulating Haspin, the inhibitors strongly reduced histone H3-Thr3 phosphorylation in quiescent cells, as evaluated by Western blotting (Figure 1c and Supplementary Information, Figure S1g for cell-cycle control) and by single-cell analysis in immunofluorescence using Ki67 as a counterstain (Figure 1d). To test this effect on another cell line and exploiting a different mean to induce quiescence, we extended our analysis to SH-SY5Y neuroblastoma cells, a model of neuronal function and differentiation [41]. Following differentiation into a neuron-like state (evaluated monitoring known differentiation markers by qRT-PCR, Supplementary Information, Figure S1h), these cells still exhibit active Haspin-targeting H3-Thr3 (Figure 1e). The results exclude any possible residual mitotic phosphorylation from the previous cycle before starvation and confirm that Haspin is active in noncycling cells, reinforcing previous findings reporting Haspin activity also outside of the M phase [42].

### 2.2. H3-Thr3 Phosphorylation Regulates Primary Cilia Length and Resorption

One key feature of most quiescent cells is the appearance on their surface of primary cilia, microtubular organelles that exert different roles from cell-cycle regulation to sensing [23,35]. In particular, the maturation of a centriole into a basal body makes it unable to sustain a mitotic apparatus, preventing cell-cycle reentry of quiescent cells [43].

**Figure 2:**
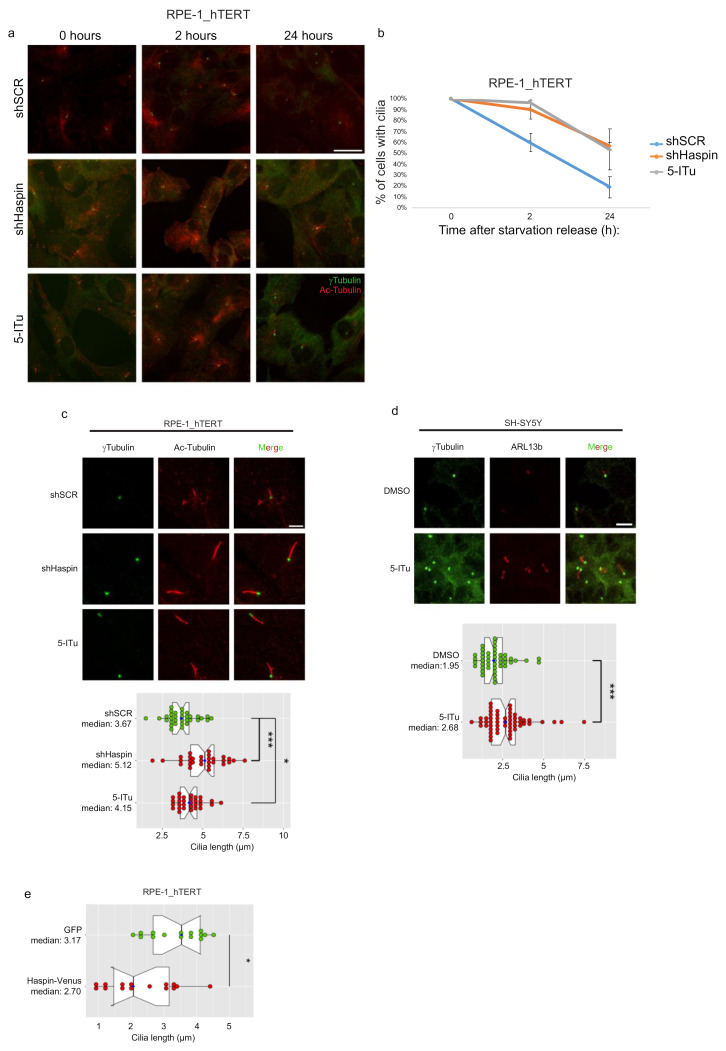
Loss of Haspin leads to longer and more persistent primary cilia. (**a**,**b**) shSCR and shHaspin were treated as described in Figure 1. For Haspin inhibition, entry into G_0_ was achieved by 48 h of serum starvation to induce ciliation. 5-ITu was added after the first 24 h of starvation. Cells were then incubated in the presence of serum to induce cell-cycle entry and cilia resorption. Samples were analyzed by immunofluorescence in G_0_, 2 h, and 24 h after serum readdition. Cells were fixed and processed for immunofluorescence against **γ**-tubulin and acetylated tubulin; representative images are shown in (**a**), and the percentage of cells with or without cilia is shown in (**b**); error bars represent standard deviation; (**c**) cells were processed as above to measure cilia length at the end of the incubation in serum-free medium. Representative images are shown; (**d**) SH-SY5Y cells were differentiated and processed by immunofluorescence to visualize basal bodies (**γ**-tubulin) and cilia (ARL13B). Representative images are shown; (**e**) RPE_1-hTERT cells were transfected with GFP or Haspin–Venus encoding plasmids and serum-starved for 48 h. At the end of the starvation, cells were fixed and processed for immunofluorescence to measure cilia length. Graphs in (**c**–**e**) show the median cilia length calculated as described in Material and Methods; boxes include 50% of the data points, notch represent confidence interval (median ± 1.58 IQR/sqrt(n)). *t*-test was applied as a statistical measurement, n.s.; not significant, * *p* < 0.05, *** *p* < 0.005. Scale bar in (**a**): 20 µm, (**c**,**d**): 5 µm.

Since we found Haspin to be active in noncycling cells, we investigated whether any relationship may exist between Haspin and primary cilia dynamics. Ciliogenesis was induced in control and Haspin-silenced or -inhibited RPE-1_hTERT cells by serum starvation. Cells were then released in a serum-containing medium to promote cilia resorption (a process essential to cell-cycle reentry). Samples were taken at the end of starvation or during resorption at 2 and 24 h after the release. The addition of serum, which triggers cell cycle reentry (Supplementary Information, Figure S2a), promotes cilia resorption in control cells, while when Haspin activity is compromised, cilia are persistent (Figure 2a,b). Moreover, we observed that loss of Haspin activity leads to a statistically significant increase in cilia length in starved cells (Figure 2c). This confirms that the role of the kinase is not restricted to reentry in the cell cycle and positions Haspin within the mechanisms that determine the length of the cilia. Similar results were obtained exploiting Haspin chemical inhibitor 5-ITu in differentiated neuroblastoma SH-SY5Y cells (Figure 2d and Figure S1h as expression levels of MAP2 and RARb gene as a differentiation control, primers used are listed in Table 1). In agreement with our conclusions, overexpression of Venus-tagged Haspin led to a significant reduction in the length of primary cilia in noncycling cells, confirming the observed inhibitory role on ciliogenesis played by Haspin (Figure 2e and Supplementary Information, Figure S2b).

**Figure 3:**
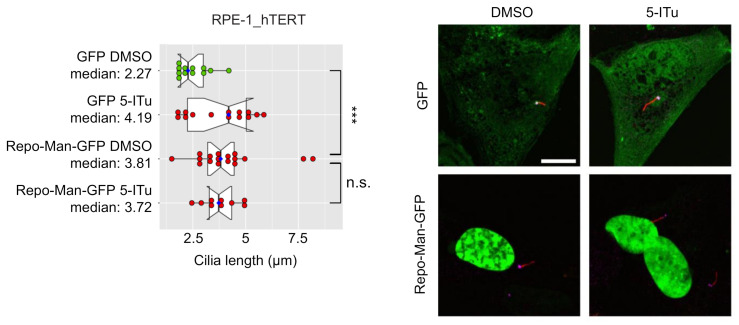
Loss of H3-Th3p causes an increase in cilia length. RPE-1_hTERT cells were transfected with a GFP- or Repo-Man-GFP- encoding plasmid and serum-starved for 24 h before being incubated, in serum-free media, with DMSO or 10 nM 5-iTU for a further 24 h. Cells were then fixed and processed for immunofluorescence against **γ**-tubulin (pink) and acetylated-tubulin (red). Representative images are shown (scale bar: 10 µm). Graph shows the median cilia length calculated as described in Material and Methods; boxes include 50% of the data points, notch represent confidence interval (median ± 1.58 IQR/sqrt(n)). *t*-test was applied as a statistical measurement, n.s.; not significant, *** *p* < 0.005.

Although being the most characterized, H3-Thr3 is not the only known target of Haspin [44,45]. To gain a first mechanistic insight on how Haspin activity regulates primary cilia dynamics, we investigated whether H3-Thr3 dephosphorylation was sufficient to reproduce the defects observed upon loss of Haspin activity. H3-Thr3 has been shown to be dephosphorylated by PP1 in a Repo-Man-dependent manner, and overexpression of Repo-Man promotes efficient H3-Thr3 dephosphorylation [2]. We overexpressed Repo-Man-GFP in RPE-1_hTERT cells and serum-starved them to induce ciliogenesis. As shown in Figure 3, Repo-Man led to the formation of a significantly longer cilium, similar to what was observed by Haspin inhibition. Remarkably, when Repo-Man was overexpressed, 5-ITu did not further affect cilia length. These observations show that Haspin inhibition is not additive to Repo-Man overexpression, emphasizing the role played by histone H3-Thr3 and excluding possible off-target effects.

### 2.3. Preventing Dido3 Nuclear Recruitment Suppresses the Cilia Defects of Haspin-Lacking Cells

Previous evidence showed that Dido3 is specifically recruited to chromatin through direct interaction with H3K4me_3_ [20]. In vitro experiments with H3 peptides showed that a variety of histone H3 modifications affected the interaction between the PHD domain of Dido3 and H3K4me_3_. In particular, H3-Thr3 phosphorylation completely abolished the binding of Dido3 to H3 in vitro and negatively correlated to Dido3 recruitment to chromatin in vivo throughout mitosis [20]. Intriguingly, Dido3 was previously reported to be critical for targeting HDAC6 to the basal body of the cilium, where it regulates cilium length and dynamics [22]. Thus, we hypothesized that the molecular mechanism underlying abnormally long cilia in the absence of Haspin activity could reside in the persistent binding of Dido3 to H3K4me_3_, due to deficient H3-Thr3 phosphorylation, depleting HDAC6 from the cilium. Our attempts to localize Dido3 and HDAC6 were unsuccessful for technical reasons; thus, we opted for a genetic approach to test this hypothesis. If the defect in cilium length and persistence caused by loss of Haspin is due to sequestration of Dido3 in the nucleus through persistent binding to H3K4me_3_, forcing Dido3 exclusion from the nucleus should restore proper deciliation and cilium length.

**Figure 4:**
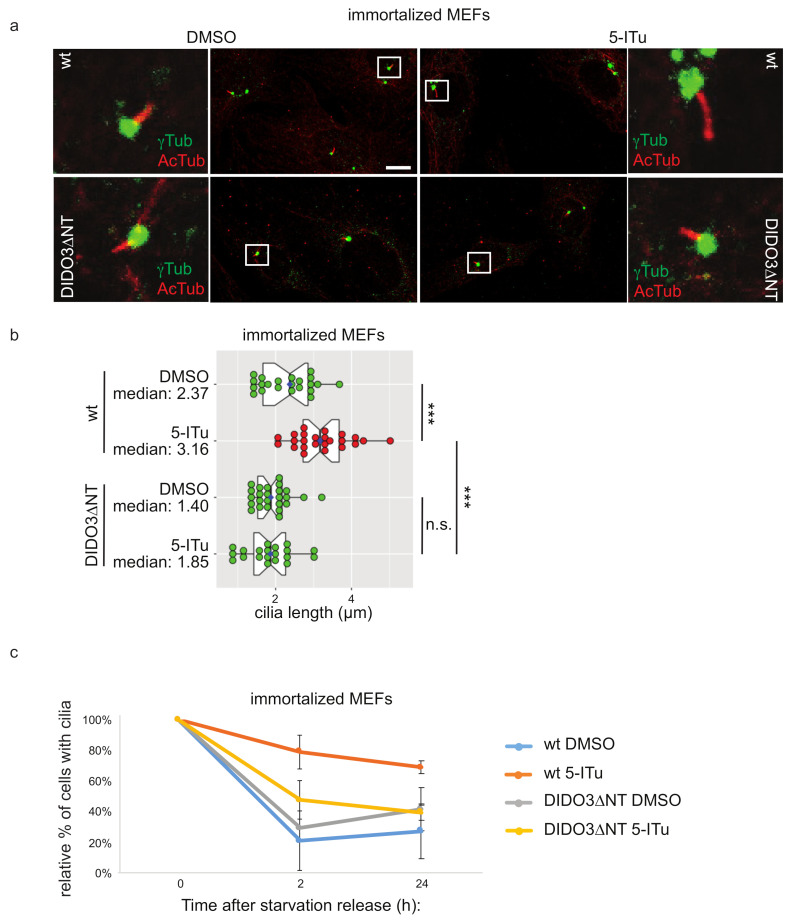
Displacement of Dido3 from the chromatin suppresses Haspin-dependent ciliary defects. Control or Dido3∆NT MEFs were driven in G_0_ by 48 h serum-starvation to induce ciliation and then treated overnight with DMSO or 10 nM 5-ITu ((**a**) and time 0 of (**c**)). At the end of the treatment, cells were incubated with a serum-containing medium to induce cilia resorption (in (**c**) for 2 and 24 h). Cilia length was measured by immunofluorescence analyzing acetylated tubulin and **γ**-tubulin (**a**). Representative images are shown in (**a**) (scale bar: 10 µm); cilia length is reported in graph (**b**); boxes include 50% of the data points, notch represent confidence interval (median ± 1.58 IQR/sqrt(n)). *t*-test was applied as a statistical measurement, n.s.; not significant, *** *p* < 0.005. Panel (**c**) shows cilia resorption kinetics represented as the normalized percentage of ciliated cells 2 h or 24 h after serum re-addition post-release. Error bars represent standard deviation.

A Dido3∆NT mutant was shown to be incapable to bind H3K4me_3_, while still effectively localizing to the basal body along with HDAC6, with a concomitant shortening of cilia in serum-starved MEFs [22]. In the same MEFs, we monitored whether the long cilium phenotype induced by Haspin inhibition could be reverted by Dido3∆NT. As shown in Figure 4a,b, Haspin inhibition in MEFs caused a significant increase in the length of the cilia. On the contrary, MEFs expressing genomic Dido3∆NT exhibited shorter cilia that were largely unaffected by 5-ITu treatment. Similarly, the delay in cilia resorption observed in wild-type MEFs after Haspin inhibition was abolished in a Dido3∆NT background (Figure 4c). Similar results were obtained in primary MEFs (Supplementary Information, Figure S3). These observations confirm that Haspin activity promotes the transfer of Dido3 from the chromatin to the basal body where it regulates cilium length and deciliation.

### 2.4. Haspin Inhibition Causes a Ciliopathic Phenotype in Zebrafish Embryos

To investigate the physiological relevance for this modulatory role of Haspin on cilium dynamics, we exploited zebrafish embryos that proved to be an excellent model to study Vertebrate ciliogenesis [46]. Zebrafish Haspin is located on chromosome 1, consists of 18 exons and 17 introns, and encodes a 1092 aa protein. RT-PCR on RNAs extracted from pools of wild-type zebrafish embryos reveals Haspin expression at all stages analyzed, starting from the very first cleavage stages (2–4 cells) to 72 hpf (hours postfertilization), showing both maternal and zygotic expression (Supplementary Information, Figure S4).

**Figure 5:**
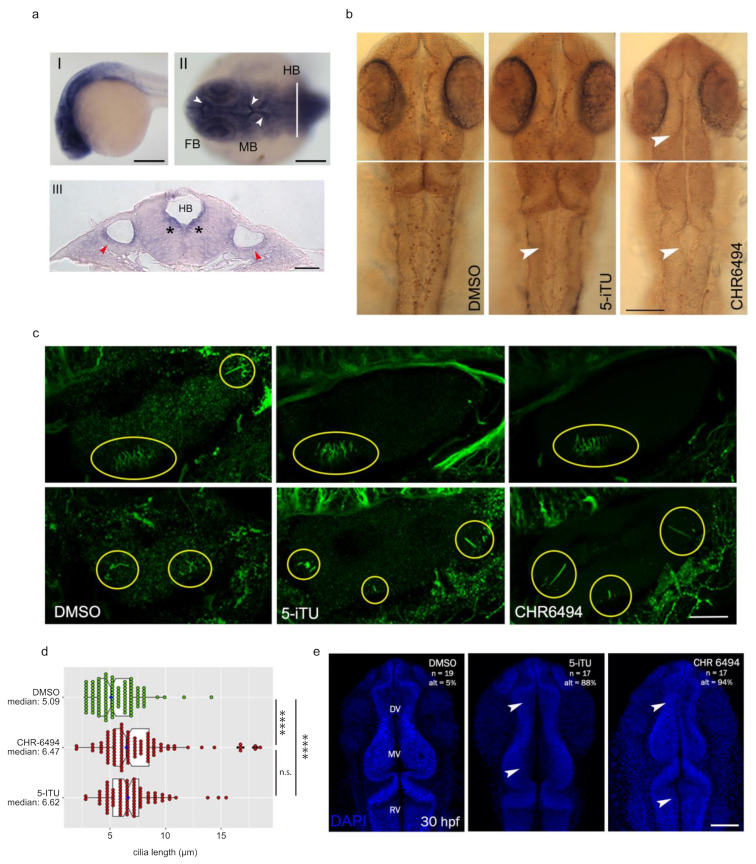
Haspin regulates cilia in the zebrafish embryo. (**a-I**) expression pattern of zebrafish Haspin at 24 hpf. Lateral view, anterior to the left. The gene is expressed in all brain vesicles; (**a-II**) dorsal view of the cephalic region, anterior to the left. The neuromeres are all heavily labeled, with a further intense signal pinpointing the whole periventricular portion of the developing brain (white arrowheads); (**a-III**) histological section conducted according to the plane shown in a-II (white line). The signal is clearly visible in the cells surrounding the ventricles (black asterisks) and the cells of the otic vesicle (red arrowheads). FB, forebrain; MB, midbrain; HB, hindbrain; (**b**) embryos were treated with either DMSO, 5-ITU, or CHR-6494 until 48 hpf, when they were processed for immunohistochemistry with a phosphorylated H3-T3 antibody. Dorsal views are shown; white arrowheads indicate points of reduced histone phosphorylation; (**c**,**d**) embryos were incubated with DMSO or Haspin inhibitors for 72 h when they were fixed and processed for immunofluorescence against acetylated tubulin. Yellow circles indicate ciliated regions, two different z stacks for each sample are shown; cilia length was measured and is reported in d; boxes include 50% of the data points, notch represent confidence interval (median ± 1.58 IQR/sqrt(n)). *t*-test was applied as a statistical measurement, n.s.; not significant, * *p* < 0.05, **** *p* < 0.001; (**e**) embryos were treated with DMSO or Haspin inhibitors for 30 h and then stained with DAPI to monitor neural tube morphology. Scale bars: (**a-I**) 200 µm; (**a-II**) 50 µm; (**a-III**) 10 µm; (**b**) 100 µm; (**c**) 25 µm; (**e**) 100 µm.

To restrict our analysis of Haspin-related phenotypes, we first determined its spatial distribution in the zebrafish embryo by whole-mount in situ hybridization, which shows Haspin expression in the cephalic region of the embryo at 24 hpf (Figure 5a). In particular, as visible in the flat-mount preparation (panel II) and the histological section (panel III) of hybridized embryos, the Haspin specific probe selectively paints anatomical districts such as the optic vesicle and the periventricular portion of the developing brain structures (red arrows and asterisks, respectively). Remarkably, both these regions are characterized by the presence of ciliated cells.

To unveil the role of Haspin in zebrafish, we treated the embryos with 5-ITu and CHR-6494 inhibitors at 10 nM and 50 nM (as well as in RPE and Sh-SY5Y cells), respectively. The embryos were treated from one-cell stage to 24 hpf, then tested for H3-Thr3 phosphorylation. The embryos were analyzed at different stages of development and treated embryos, compared to controls, showed a drastically reduced number of phosphorylated H3Thr3 positive cells, at 48 hpf (Figure 5b), thus strictly replicating the cell lines behavior in a condition of Haspin deficiency.

We then exploited Haspin chemical inhibitors to look for cilia alterations in the zebrafish embryo. As mentioned above, *haspin* is expressed in all the developmental stages analyzed (from one-cell stage to 72 hpf). In order to directly characterize the effects of Haspin deficiency on cilia development and maintenance, we analyzed the inner hair cells cilia of 72 hpf larvae (Figure 5c). Indeed, at this stage, the inner ear is characterized by the presence of sensory patches in which the cells display, on their apical surface, a specialized cilium, named kinocilium. This microtubule-based structure is therefore easily detectable by means of immunofluorescence employing an antibody directed against acetylated tubulin [47]. The length of the kinocilia has been measured in four different areas of the ear. In agreement with the results obtained from cell cultures, Haspin inhibition leads to a statistically significant increase in cilia length in zebrafish embryos. No significant differences were observed comparing 5-ITu to CHR-6494 embryos (Figure 5d).

In the ventricular region, the cilia are correlated with the cerebrospinal fluid (CSF) flow [48,49], which is at the basis of proper development of the brain and spinal cord [50]. Therefore, impaired cilia functionality could cause developmental impairments in the neural tube. Indeed, embryos treated with both inhibitors showed profound alterations of the brain ventricles (Figure 5e). In the 87% (*n* = 20) of the 5-iTU and the in 94% (*n* = 20) of the CHR6494 treated embryos, diencephalic/mesencephalic (DV/MV) and rhombencephalic (RV) ventricles lumens resulted compromised, with ventricles appearing either enlarged or collapsed in comparison to controls. These effects could be associated with the alterations in cilia formation/maintenance/function along the ependymal ventricular walls [51] due to lack of Haspin activity.

## 3. Discussion

Posttranslational modifications of histones are widely exploited by the cells to create a layer of epigenetic regulation of gene expression as well as to recruit protein to the chromatin in a transient and specific way during the cell cycle or in response to specific stimuli [52]. Among all histones, H3 is the most subjected to PTMs.

Phosphorylation of H3 on threonine 3 represents a remarkable event in the mitosis of eukaryotic cells. Indeed, this PTM, together with Bub1-mediated phosphorylation of H2A on serine 121, directs the recruitment of the chromosomal passenger complex to inner centromeres, allowing a proper alignment of chromosomes on the metaphase plate [5]. However, this is not the only role for H3-Thr3 phosphorylation, which has been shown to drive asymmetric histone inheritance [13], chromosome cohesion [11,53], and regulate chromatin architecture [42]. Less characterized aspects of H3-Thr3 phosphorylation are the consequences of the displacement of proteins that bind the surrounding residues when H3-Thr3 is not phosphorylated. Indeed, several proteins bearing a PHD domain are recruited to H3-K4me3 but are dispersed upon H3-Thr3 phosphorylation [17,20].

Haspin kinase, responsible for targeting H3-Thr3, has long been regarded as only active throughout mitosis, owing also to an autoinhibitory domain which is displaced by other mitotic kinases [38]. Here, we show that Haspin phosphorylates histone H3 also in quiescent and differentiated cells. These results integrate recent evidence suggesting that Haspin is active in cycling cells also outside mitosis [42], implying a more complex mechanism underlying its regulation than previously thought [38].

Noncycling cells are intrinsically not susceptible to phenotypes induced by chromosome mis-segregation. Thus, our finding that in postmitotic cells H3-Thr3 is phosphorylated by Haspin, albeit to a lower extent, compared to mitotic cells, provided an ideal tool to dissect the impact of Haspin kinase in a chromosome-segregation-independent context. We showed that a major role for H3-Thr3 phosphorylation in noncycling cells is represented by the regulation of cilia length and resorption. The new role for Haspin-dependent H3-Thr3 phosphorylation in controlling cilia elongation and stability reveals the existence of a signaling system transducing a nuclear stimulus into a ciliary response. We then searched for proteins that could be re-localized from the chromatin to the cilium upon H3-Thr3 phosphorylation. Previous reports suggested that PHD-containing proteins, in particular Dido3, would be ideal candidates. Indeed, Dido3 binds H3-K4me3 but the binding is prevented by H3-Thr3 phosphorylation [17,20]. Moreover, Dido3 directs the cellular localization of the histone deacetylase HDAC6, which in turn drives the shortening of the cilia [22]. We found that in MEF-expressing Dido3∆NT, a mutant that cannot bind to H3K4me3 but retains recruitment at the basal body, Haspin inhibition did not impact cilia length or persistence, proving the involvement of Dido3 and HDAC6 in an Haspin-dependent mechanism to modulate cilia dynamics. When Haspin is inactive, H3-Thr3 is not phosphorylated, Dido3 stably remains at chromatin through H3-K4me_3_ binding, along with HDAC6, and cilia elongation is no longer under control.

By exploiting the zebrafish embryo, we were able to track the physiological outcome of the deficiency in Haspin activity and deregulated primary cilia elongation. We show that the results obtained in cell lines can be recapitulated in this model. Interestingly, following Haspin inhibition, we were able to induce clear alteration in cilia morphology. Indeed, loss of Haspin activity resulted in longer kinocilia in the hair cells of the inner ear, supporting the notion that, as we have previously shown in the in vitro setting, Haspin could modulate ciliogenesis also in vivo. Moreover, impairment in Haspin activity resulted in overt defects affecting the brain ventricles, which appeared abnormally enlarged or collapsed. These phenotypes could be explained as the consequence of alterations in the development and/or functioning of the cilia along the ependymal ventricular walls due to lack of Haspin; being such makes cilia responsible for the proper cerebrospinal flow, which is vital for the correct development of the brain and spinal cord [50].

## 4. Materials and Methods

### 4.1. Cell Culture

RPE-1_hTERT cells were grown in DMEM: F12 1:1 supplemented with 10% FBS and penicillin/streptomycin (Thermo Fischer Scientific, Moscow, Russia); HEK293T, MEFs, and SH-SY5Y were grown in DMEM supplemented with 10% FBS and penicillin/streptomycin. To promote entry into G_0_ by serum starvation, RPE-1_hTERT and MEFs were incubated for 48 h in media without FBS. Differentiation of SH-SY5Y was performed as follows: 72 h in DMEM 10% FBS 15µM retinoic acid, 24 h in DMEM 3% FBS 15 µM retinoic acid, 48 h in DMEM 0.5% FBS 100µM BDNF. All cells were incubated at 37 °C and supplemented with 5% CO_2_.

### 4.2. Chemical Inhibitors

5-Iodotubercidin (I1000) and CHR-6494 (SML0648) were purchased from Sigma Aldrich Merck KGaA, Darmstadt, Germany.

### 4.3. Lentiviral Vectors Production

Haspin-targeting shRNA, shHaspin (5′-GCTGATAACAAATGTTCTGAA-3′), was inserted in a pLKO.1-TCR cloning vector (Addgene plasmid #10878) through digestion using EcoRI and AgeI, using shSCRAMBLE (Addgene plasmid #1864) as a control. We produced lentiviral vectors in HEK293T cells through cotransfection of these plasmids and packaging plasmids (pPAX2 and pMD2.G from Addgene#12259-#12259). Then, 48 h after the infection, we collected viruses in the presence of 10 mg/mL of polybrene.

shRNA

shSCR = 5′-

CCTAAGGTTAAGTCGCCCTCGCTCGAGCGAGGGCGACTTAACCTTAGG-3′;

shHaspinUTR = 5′-

CCGGGCTGATAACAAATGTTCTGAACTCGAGTTCAGAACATTTGTTATCAGCTTTTTG-3′.

### 4.4. Total RNA Extraction, Retrotranscription, and qRT-PCR Assay

Total RNA was isolated using a RNeasy Mini kit (Qiagen s.r.l., Milano, Italy) and 1 μg was retrotranscribed using iScript Reverse Transcription Supermix (Bio-Rad laboratories s.r.l., Milano, Italy) according to the manufacturer’s protocol.

cDNA was diluted 1:100 and 5 µg were used as a template for real-time PCR (2x Mastermix Sybr Green from Genespin s.r.l. was used, Milano, Italy). Analysis was performed with Bio-Rad laboratories s.r.l., Milano, Italy.

### 4.5. Cell Transfection

Cells were transfected at the beginning of the starvation period (RPE-1_hTERT) or at the end of differentiation (SH-SY5Y); plasmids for overexpression were transfected using lipofectamine 3000 following the provider’s manual (Thermo Fisher Scientific, Moscow, Russia); siRNAs were transfected using RNAiMAX (Thermo Fisher Scientific, Moscow, Russia) following provider’s instruction.

### 4.6. Western Blotting

Cells were lysed in Laemmli buffer; samples were then boiled for 10 min and sonicated. After SDS–PAGE and transfer to a nitrocellulose membrane, filters were blocked with 5% milk in PBS TWEEN-20 0.1% (PBST) and then incubated with a given primary antibody. Filters were washed 3 times in PBST, incubated 1 h at RT with secondary HRP-conjugated antibody, and washed for further 3 times.

ChemidocTouch (Bio-Rad laboratories s.r.l., Milano, Italy) was used to acquire images.

### 4.7. Antibodies

GFP (Clontech, Mountain View, CA, USA, 632677), H3 (Abcam plc., Cambridge, UK, ab1791), Haspin (Abcam plc. ab226222), Vinculin (Sigma-Aldrich V9131), HDAC6 (Abcam plc. ab1440), Dido3 (Merck-Millipore, Burlington, MA, USA, ABC480), acetylated tubulin (Sigma-Aldrich T7451), gamma-tubulin (Sigma-Aldrich T5192), Ki-67 (Thermo-Fisher Scientific SolA15), H3 Thr3 phosphorylated (Upstate 05-746), AlexaFluor goat anti-mouse 488 (Thermo-Fisher Scientific A28175), AlexaFluor goat anti-rabbit 488 (Thermo-Fisher Scientific A11034), AlexaFluor goat anti-mouse 594 (Thermo-Fisher Scientific A11032), AlexaFluor Goat anti-mouse 647 (Thermo-Fisher Scientific A21235).

### 4.8. Immunofluorescence

Cells were seeded on coverslips and treated as described above. At the end of the treatment, cells were washed 3 times with PBS and fixed for 20 min with 3.7% formaldehyde in PBS. After 3 washes with PBS, cells were permeabilized with ice-cold methanol for 20 min and blocked in 10% BSA in PBS for 1 h at RT. Primary antibodies diluted in PBST (PBS with 0.1% Tween-20) were then added and incubated overnight (for γ-tubulin) or for 2 h (for other primary antibodies). After 3 washes in PBST, cells were incubated with secondary antibodies diluted in PBST for 1 h at RT, washed 3 times in PBST, and slides were mounted using ProLong Gold with DAPI. The acquisition was performed using a Leica DMRA2 (widefield) or Nikon A1R (confocal).

### 4.9. Image Quantification

For H3-Thr3p quantification, nuclear ROIs were extracted from DAPI images and used to quantify the signal from phosphorylated-H3-Thr3. The cellular background was then subtracted to obtain the normalized fluorescence intensity. Cilia length was determined from confocal z-stacks measuring cilia length on the xy plane on a maximum projected image and on the *z*-axis multiplying the number of stacks a cilium was detectable for the z-step. Cilia were approximated as linear structures, and their length was determined by Pythagora’s theorem. All analyses were performed with FIJI [54].

### 4.10. FACS Analysis

Cells were detached through trypsinization, washed in PBS, and fixed in 70% ice-cold ethanol. Samples were then washed with PBS/BSA and stained with 20 μg/mL propidium iodide (Sigma-Aldrich P4864), 10 μg/mL RNase A (Sigma-Aldrich R6513) at room temperature for 30 min. FACS analyses were performed on a BD FACScan and quantified with Cell Quest software (BD Biosciences-EU).

### 4.11. Proliferation Assay

Cells were seeded in 96-well plates at a density of 2000 cells per well. Viable cells were assessed by MTS assay (Promega italia s.r.l., Milano, Italy) every 24 h for 4 days at 490 nm (EnSight microplate reader from PerkinElmer Inc., Waltham, MA, USA).

### 4.12. Zebrafish

Zebrafish embryos/larvae of the AB strain, obtained through natural spawning of wild-type adult fish, were raised at 28 °C in 0.002% Methylene Blue and staged as previously described [55]. In order to avoid pigmentation, 0.003% PTU (1-phenyl-2-thiourea) was added to the embryos’ water.

All procedures here described were performed in accordance with the relevant guidelines and regulations. Our facility strictly complies with the relevant Italian laws (Legislative Decree No. 26/2014), as also confirmed by the municipality of Milan (PG 384983/2013).

### 4.13. Whole-Mount In Situ Hybridization

Whole-mount in situ hybridization (WISH) experiments were performed as described [56] employing embryos and larvae previously fixed in 4% paraformaldehyde (PFA)/phosphate-buffered saline (PBS). Then, they were rinsed with a PBS-Tween solution, dehydrated in 100% methanol, and stored at −20 °C until processing. An 1166-bp Haspin fragment obtained by RT-PCR using has probe for 5′-AGTTGGAGCCTTGGATCTCC-3′ and has probe rev 5′-GGCAGTCCTCTCTTCCTGTT-3′ primers were cloned into the pGemT-Easy vector (Promega italia s.r.l., Milano, Italy). Sense and antisense RNA probes were transcribed using T7 and SP6 RNA polymerase (Roche), respectively, on templates linearized with either *SalI* or *NcoI* (New England Biolabs Inc, Ipswich, Massachusetts, USA). Riboprobes were labeled with digoxigenin using the “DIG-RNA Labelling Kit” (Roche). Images were acquired with a Leica DFC450C digital camera and the Leica Application Suite (LAS) software (Leica) on a Leitz DM RB microscope.

### 4.14. Immunohistochemistry and DAPI Staining

Whole-mount immunohistochemistry (IHC) assays were performed according to routine protocol [57]. Following the overnight fixation in 4% paraformaldehyde/PBS, embryos/larvae were washed with PBS/Tween^®^-20/TritonTMX-100, then incubated with 5% BSA (bovine serum albumin—Merck), Phospho-Histone H3 (Thr3) (Upstate 05-746) primary antibody and anti-acetylated α-tubulin (T7451, Merck). Secondary antibodies used were Dako Envision anti-mouse (cat# K4000) and goat anti-mouse Alexa fluorTM 488 (Thermo Fisher). 4′,6-diamidino-2-phenylindole (DAPI) allowed the visualization of the cell nuclei. A Leica DFC450C digital camera mounted on a Leitz DM RB microscope was used to acquire all brightfield images, employing the Leica Application Suite (LAS) software (Leica). A laser-scanning confocal microscope Nikon A1R was used to acquire immunofluorescent images.

## Figures and Tables

**Table 1:** Primers used to monitor SH-SY5Y differentiation by RT-PCR.

Target	Forward 5′-3′	Reverse 5′-3′
RAR beta	TCGATGCCAATACTGTCGACTCCA	AGCTGGCAGAGTGAAGGGAAAGT
MAP2	CATGGGTCACAGGGCACCTATTC	GGTGGAGAAGGAGGCAGATTAGCTG
GAPDH	ATGGGTGTGAACCATGAGAAG	AGTAGAGGCAGGGATGATGT

## Data Availability

Data is contained within this article or its Supplementary Material.

## Supplementary Materials

The following are available online at https://www.mdpi.com/article/10.3390/ijms22147753/s1.
