# Supplementary material for: Phosphorylation of H3-Thr3 by Haspin Is Required for Primary Cilia Regulation
Source: Int J Mol Sci. 2021 Jul 20;22(14):7753. doi: 10.3390/ijms22147753 (PMC8307231; doi:10.3390/ijms22147753)
Supplement: Supplementary file 1 [file ijms-22-07753-s001.zip › ijms-1290869-supplementary.pdf]

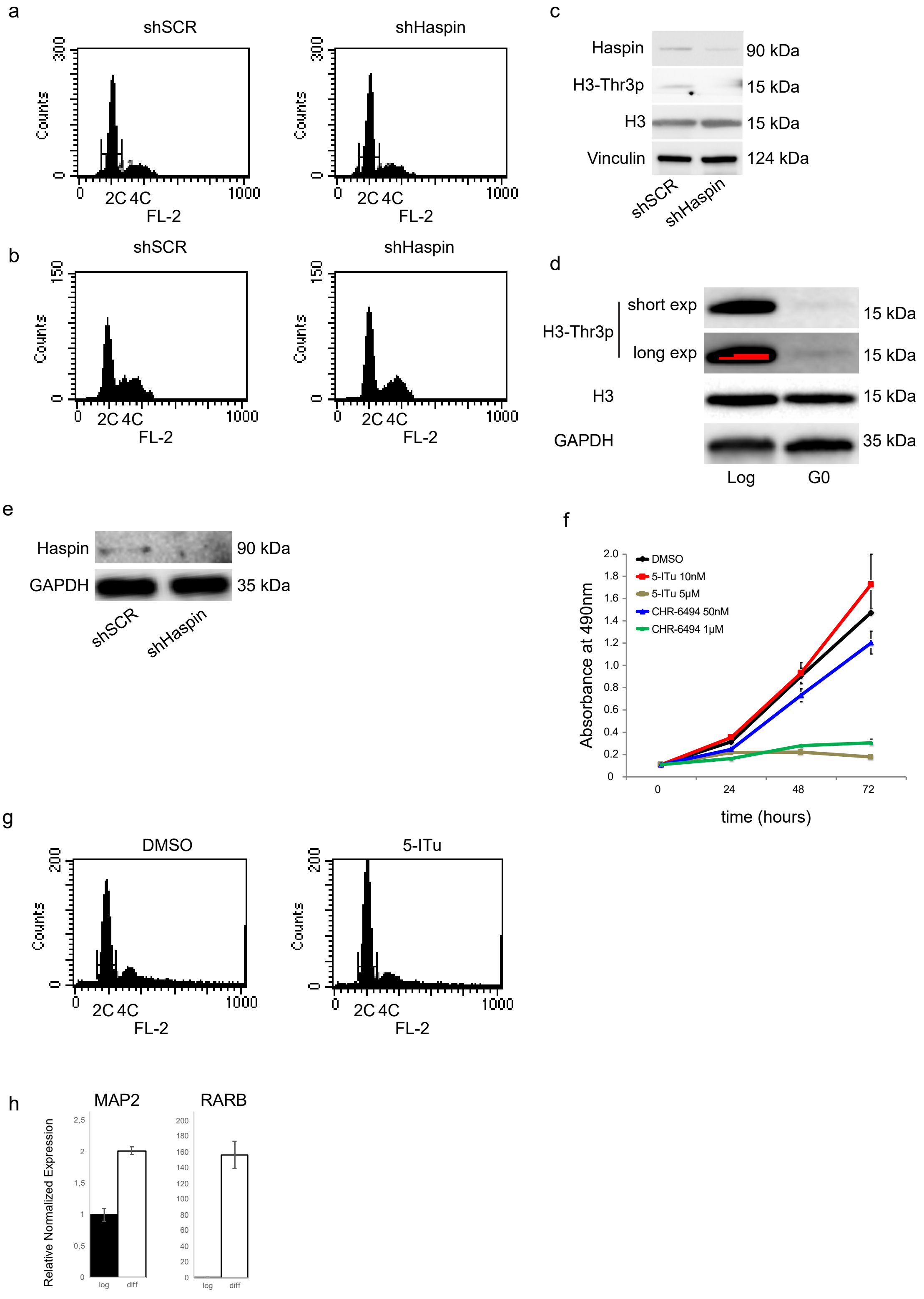

Supplementary information, Figure S1. (**a**) Cell-cycle analysis of control or Haspin-silenced RPE-1_hTERT cells after 48 hours serum starvation; (**b**) cell-cycle analysis of control or Haspin-silenced logarithmically

growing RPE-1_hTERT cells and relative Western blot; (**c**,**d**) protein extracts from either exponentially growing or serum-starved RPE-1_hTERT cells were collected and analyzed by SDS–PAGE and Western blot; (**e**) silencing control for experiment in Figure 1b; (**f**) exponentially growing RPE-1_hTERT cells were treated with different dosages of haspin inhibitors 5-ITu and CHR-6494. The graph shows a growth curve measured by MTS assay from two independent experiments; (**g**) cells were treated as in Figure 1C and cell cycle was monitored by flow cytometry; (**h**) relative abundance of differentiation markers (MAP2, RARB) measured by RT-PCR of cells in experiment 1e, showing the efficacy of the differentiation protocol.

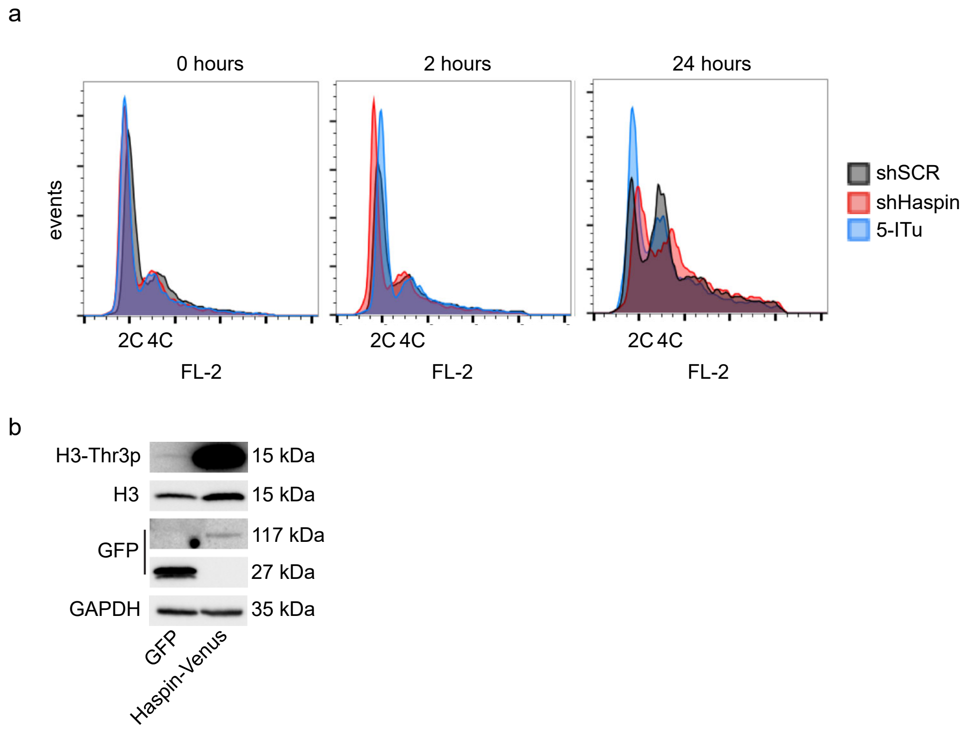

Supplementary information, Figure S2. (**a**) Cell-cycle analysis of shSCR, shHaspin, and Haspin-inhibited cells from experiment in Figure 2b; (**b**) protein extracts from cells treated as in Figure 2e were analyzed by SDS–PAGE and Western blot to monitor H3-T3p abundance.

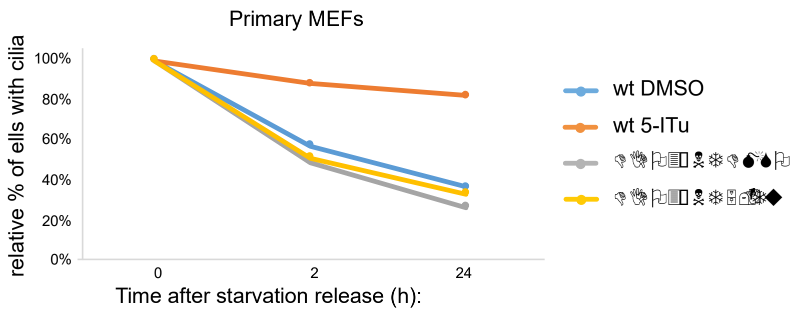

Supplementary information, Figure S3. Primary MEFs of the indicated genotype were treated as in Figure 3c; the graph shows the relative percentage of ciliated cells at the indicated time points.

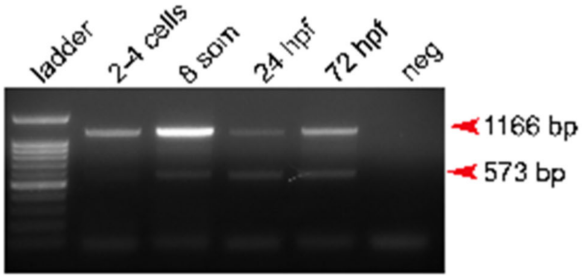

Supplementary information, Figure S4. Ethidium bromide-stained gel of a qualitative RT-PCR performed with the zhaspPCR frw: 5′-GCTGCTAATGAATGTGCTGG-3′ and zhaspPCR rev: 5′-CTCTTCCTGTTTCTTGCTGC-3′ primers using few selected stages representative of the whole developmental window analyzed, and with no cDNA as negative control (neg). Red arrowheads indicate the 1166 and 573 bp bands, which represent the two products of alternative splicing of the *hsp* hnRNA. The mRNA is present starting from the very early stages (2-4 cells) to 72 hpf, supporting the notion that *hsp* expression is both maternal and zygotic.
